# Udiometrical, Thermometrical, and Barometrical Statements

**Published:** 1810-03

**Authors:** T. Pole

**Affiliations:** Bristol


					I diomdricaL Tkefmometrical,and Barometrical Statements$
by Dr. T. Pole, of' Bristol.
An Account af the Quantity of Rain fallen in each Month.
O .
fc-. ??
O G
O
13 S
CJ
0
1
Ic
Months, as de-
nominated in
the Calendar.
1804
1805
"o o
C O
1806
QJ c
"o ?
c o
1807
180S
? 2
C o
1809;
. ^3
u ?
.s
e
in
O o
O o
C O
1 !
2
3
4
5
?
7
8
9
10
n
12
January -
February -
March
April - -
May - -
June - -
(July - -
August
September
October
November
December
43
48
SO
27
75
0 25
3 78
2 26
0 28
2 80
5 44
1 45
Total [29 77|2tf
44
30|
9S
78
43
58
6'0
22
59
94
32
73
1
97
14! 2
67
29:
50
32
87
2 7
81
49
36
39
28
15
34
49
82
15
21
55
6'9
14
44
38|31 31
5
591
35
37
99
75
76
6
36
261
8
52
12.
26*
27
75
45
75
7
38
16
S
54"
68
32 8|29 51
The average Temperature of each Month, from Observations mads
at 8 in the Morning.
o "5
c
3
4
5
6"
7
8
.9
10
11
12
Mouths, as de-
nominated in
the Calendar,
1804- 1805
O cn
o jz,
J??
Q
January
February -
March
April - -
May - -
J une - -
July
August
September
October
November
December
33 50133
3o 33:35
33 10
9942
? 57
?j57
k>2 ? 6' I
;6o 33.63
1SC6
fcfiQ
c 2
16137
86)37
20(37
87
30'
50! 61
6*3
' /
75
9A
43 80
54 1?
80
33
56 32 58 ?
49 99,43 ?
42 1036 ?
3-3 50/37 ?
6'2 2 2
54 52
48 66
45 30
44 44
1S07
tPc
QS
1808
C -
31 o 'S
35 75
33 46"
42 33
55 66
59 45
64 44
63 52
48 27
51 46
34 55
31 55
33 47
34 15
33 66
43 10
[06 90
59 90
66
1809
^ o
33 17
+2 11
30 56
39 77
56 73
58 85
61
\61
63
45
44
42 521.36
33 10J37
90
44
56 76"
US 17
The
256 Dr. Poli's Tubh'S of Temperature, <Sfc.
The average State of the Barometer for each Month.
f js
o c
? o
48 ?
u ?"
X ?
o -a
E "
vu c
]
2
3
4
5
6
7
8
9
10
n
12
Months, {is de-
nomirtatcc} in
the Calendar.
January ?
February ?
March
April - ?
May
[June - -
July - -
August
September
October -
November
December
1S041 18051 1800 ISC? lSOSl 180p
v o
"5 o
c O
29 60
30 25
29 70
29 SO
29 40
30 35
?29 9'
30 ?
0 65
29 75
29 90
?29 S3
o O
G O
12
<U C
-g o
G C
O C
1 c
29
29
30
'29
30
30
30
30
30
29
30
29
6o
85
90
29
30
29
29
30
30
29
30
30
9
2.9
80|30
62
5
50
90
5
20
90
10
90
85
20,
30
29
30
30
30
30
30
29
29
3 O
29
30
5
91
15
30
95
. 7
15
75
90
10
75
15!
30
30
30
30
30
30
30
29
9
29
29
29
10|
5
30
15
15
25
25
45
50
$5
6o
60
|?
3o~n
30 75
29 58T
30 30"
31 10
31 30
31 42
31 49
31 40
31 4
31 10
29 13
The highest Temperature of the Atmosphere, indicated by the"
Thermometer, at any one Time during the Summers
of the last Six Years.
C -2
^ ?
Months, as de-
nominated in
the Calendar,
June - -
[July - -
September
June - -
July - -
July - -
7 lJuly
?
c ~
s 5
CL>
CJD
o
o
Years.
8 6
86
77
S'4
85
.91
78
1804-
1805
1806
180/
180S
1803'
The*
The lowest Temperature of the Atmosphere,- indicated by- the
Thermometer, on the two coldest Days in the last Six Years,
according to Observations made at Eight o'Clock in th? Morri-
?O
t-B
o c
_ o
o ~
?Z. o
3
12
2
11
1
10
1
Months, as de-
nominated in
the Calendar.
March
December
February
November
[January -
October -
January -
12 [December
1 January -
12 jDecember
1 January -
11 jNovembcr

				

